# Sodium-Glucose Cotransporter 2 Inhibitors in Alport Syndrome: Emerging Clinical Evidence and Mechanistic Insights

**DOI:** 10.34067/KID.0000001166

**Published:** 2026-05-28

**Authors:** Kana N. Miyata, Jeffrey H. Miner

**Affiliations:** 1 Division of Nephrology, Saint Louis University, St Louis, Missouri; 2 Division of Nephrology, WashU Medicine, St Louis, Missouri

**Keywords:** Alport syndrome, CKD, fibrosis, glomerular disease, proteinuria, SGLT2 inhibitors

Alport syndrome (AS) is an inherited kidney disease caused by pathogenic variants in type 4 collagen genes (*COL4A3*, *COL4A4*, and *COL4A5*), resulting in structural defects of the glomerular basement membrane. These abnormalities initiate a cascade of podocyte stress and detachment, progressive proteinuria, and activation of downstream inflammatory and fibrotic pathways within the tubulointerstitium. Over time, these maladaptive responses drive irreversible kidney scarring and loss of function, culminating in ESKD.

At present, renin-angiotensin-aldosterone system inhibitors (RAASi) with angiotensin-converting enzyme inhibitors (ACEi) or angiotensin receptor blockers remain the only established standard-of-care therapy for AS. Sodium-glucose cotransporter 2 inhibitors (SGLT2i) have demonstrated robust kidney-protective effects in nondiabetic CKD, including glomerular diseases, but the evidence specific to AS remains limited. Moreover, the mechanisms of SGLT2i-mediated nephroprotection remain incompletely understood, including whether their effects are limited to renal tubules, where SGLT2 is primarily expressed, or extend to direct actions on glomerular cells. Proposed mechanisms include restoration of tubuloglomerular feedback with consequent reduction in intraglomerular pressure and glomerular hyperfiltration as well as anti-inflammatory and antifibrotic effects within tubular epithelial cells.

In this issue of *Kidney360*, Zheng *et al.*^1^ address this important knowledge gap by providing compelling mechanistic and translational evidence supporting the use of SGLT2i in AS in both mice and humans. In a cohort of 21 patients with AS treated with dapagliflozin, the authors report a 29% reduction in proteinuria after a mean follow-up of approximately 12 months, with preservation of eGFR. Complementary mechanistic studies in *Col4a3* mutant mice demonstrated that dapagliflozin attenuated kidney injury and suppressed the activation of immune-related pathways, as revealed by RNA sequencing of the renal cortex.

Notably, the authors identified inhibition of the stimulator of IFN gene (STING) pathway as a key mechanism underlying dapagliflozin-mediated suppression of the proinflammatory phenotype in *Col4a3* mutant tubular epithelial cells.^1^ STING signaling has been implicated in a range of kidney diseases, including AKI, diabetic kidney disease, and glomerular diseases.^2^ In these conditions, cytosolic DNA activates cyclic guanosine monophosphate-AMP synthase-STING signaling, triggering downstream inflammatory and fibrotic responses in renal cells. Previous studies of cyclic guanosine monophosphate-AMP synthase-STING signaling in AS have largely focused on podocytes.^2^ Therefore, this work expands the current paradigm by highlighting a pathogenic role for STING activation in renal tubular cells as part of AS pathophysiology.

This new study also complements prior experimental investigations of SGLT2i in AS. As summarized in Table 1, four studies have evaluated SGLT2i across different AS mouse models, yielding heterogeneous results. One study reported improved renal function and survival with SGLT2i monotherapy, whereas the addition of SGLT2i to RAASi did not confer incremental benefit beyond RAASi alone.^3^ Another study similarly found no survival advantage of combined therapy compared with RAASi alone.^4^ By contrast, two studies demonstrated that while SGLT2i monotherapy failed to improve renal outcomes, combination therapy with RAASi resulted in superior preservation of renal function, improved renal histology, and enhanced survival compared with RAASi alone.^5,6^ Mechanistically, SGLT2i effects have been linked to reduced lipid droplet accumulation and apoptosis in podocytes^3^ as well as attenuation of tubular cell senescence and the senescence-associated secretory phenotype.^5^

**Table 1:** Experimental and clinical studies of sodium-glucose cotransporter 2 inhibitors in Alport syndrome

References, Yr	Animal Model	Intervention	Main Results
**Experimental studies of SGLT2is in AS mouse models**			
Ge, *et al.* 2023^3^	Male and female *Col4a3*^−/−^ mice, 129X1/SvJ background	Empagliflozin-supplemented chow (70 mg/kg) initiated at 4 wk of age	Empagliflozin monotherapy extended lifespan in AS mice
			Reduced renal lipid accumulation and improved renal function
			Addition of empagliflozin to ramipril did not confer additional renoprotection
			Reduced lipid droplet accumulation and apoptosis in AS podocytes by empagliflozin *in vitro*
Zhu, *et al.* 2023^4^	Male and female *Col4a3*^−/−^ mice, 129/SvJ background	Food admix: (*1*) vehicle, (*2*) ramipril (10 mg/kg), (*3*) ramipril+empagliflozin (30 mg/kg), or (*4*) ramipril+empagliflozin + finerenone (10 mg/kg), starting from 6 wk of age	Mean survival: 63.7±10.0 d (vehicle), 77.3±5.3 d (ramipril), 80.3±11.0 d (ramipril+empagliflozin), and 103.1±20.3 d (triple therapy)
Miyata, *et al.* 2025^5^	Male and female *Col4a3*^−/−^ mice, 129S1/SvlmJ background	Dapagliflozin (1.5 mg/kg per d), ramipril (10 mg/kg per d), or both (D/R) *via* drinking water from 4 wk of age	Dapagliflozin monotherapy did not improve renal function or survival
			At 15 wk, D/R-treated mice showed better renal function and histopathology than those on ramipril alone
			D/R extended survival compared with ramipril alone
			D/R-treated mice exhibited reduced lipid accumulation and cell senescence in renal tubules
Horizono, *et al.* 2025^6^	Male *Col4a5<tm1Yseg>*G5X mutant, C57BL/6 background	Dapagliflozin, losartan (weak antiproteinuric effect), olmesartan (strong antiproteinuric effect), or dapagliflozin combined with each ARB, starting at 6 wk of age	Dapagliflozin enhanced renoprotection when combined with losartan but not with olmesartan
			Losartan and olmesartan alone, but not dapagliflozin, extended survival
			Survival with olmesartan+dapagliflozin was comparable to that of olmesartan alone
Zheng, *et al.* 2026^1^	Male *Col4a3* p.C1615Y transgenic mice, 129S2/Sv background	Dapagliflozin (10 mg/kg per d) *via* drinking water for 28 days, starting at 17 weeks of age	Dapagliflozin attenuated tubular injury
			Dapagliflozin reduced pro-inflammatory cytokines and profibrotic genes expressions in the renal cortex
			*Col4a3* mutant tubular epithelial cells were more susceptible to inflammatory stimuli than wild-type cells
			STING signaling was activated in renal tubules of AS mice and suppressed by dapagliflozin

Study, Reference, Yr	Patient Characteristics	Use of RAASi (%)	Follow-up Period	Main Results
**Clinical studies of SGLT2is in AS**				
Case series, Boeckhaus, *et al.* 2021^7^	Four patients, XLAS	75	3–4 mo	Two patients had a reduction in proteinuria
				Two patients did not demonstrate short-term benefit
Observational study (NCT02378805) Boeckhaus, *et al.* 2024^9^	112 (including ten children), XLAS (66%), autosomal AS (33%), digenic heterozygous *COL4A3*/*COL4A5* variants (1%)	93	Up to 32 mo	Albuminuria decreased by >30% at follow-up visits (1–3, 4–8, and 9–15 mo)
				Mean eGFR decline: 9±12 ml/min per 1.73 m^2^
Case report, Agrawal, *et al.* 2025^8^	One patient, heterozygous *COL4A4* variant	100	1 yr	Proteinuria reduced from 1246 mg/d to 1007 mg/d
Single-arm, prospective study, Zheng, *et al.* 2026^1^	21 patients, seven ADAS, two ARAS, and 12 XLAS	100	1 yr	24 h urine protein decreased by 29%
				No significant change in eGFR

ADAS, autosomal dominant AS; ARAS, autosomal recessive AS; ARB, angiotensin receptor blocker; AS, Alport syndrome; D/R, dapagliflozin plus ramipril; RAASi, renin-angiotensin-aldosterone system inhibitor; SGLT2i, sodium-glucose cotransporter 2 inhibitor; STING, stimulator of IFN genes; XLAS, X-linked AS.

Clinical evidence remains limited but is emerging. In addition to a case report and small case series,^7,8^ an observational study of 112 patients with AS demonstrated a >30% reduction in albuminuria following the addition of SGLT2i to RAASi in 93% of patients.^9^ This study by Zheng *et al.* represents the second largest reported AS cohort treated with SGLT2i to date (*N*=21) and similarly demonstrates a clinically meaningful reduction in albuminuria without significant safety concerns. Collectively, these findings support the potential role of SGLT2i as adjunctive therapy for patients with AS who have persistent albuminuria despite optimized renin-angiotensin-aldosterone system inhibition. Nonetheless, adequately powered prospective trials with placebo control and longer follow-up are essential to determine whether albuminuria reduction translates into sustained preservation of kidney function over time. The ongoing Dapagliflozin in CKD in Adolescents and Young Adult Patients Alport trial, a multicenter, randomized, double-blind, placebo-controlled study, evaluating dapagliflozin in pediatric and young adult patients with AS, will be particularly informative in addressing this question.^10^

There are several limitations to note for this study. First, while the mechanistic investigations focused predominantly on renal tubular pathways, the principal clinical outcome in the human cohort was reduction in albuminuria, a marker traditionally associated with glomerular injury. The mechanistic link between decreased tubular inflammation and immune cell infiltration and the observed reduction in albuminuria was not fully delineated. Second, given the substantial heterogeneity in disease progression across AS genotypes, larger cohorts with genotype-stratified analyses of renal outcomes would strengthen the clinical relevance of these findings. Third, as acknowledged by the authors, the precise molecular mechanism by which dapagliflozin inhibits STING signaling remains unexplored.

Despite these limitations, the clinical and pathophysiologic implications of this study are significant. Zheng *et al.* not only provide further support for the therapeutic potential of SGLT2i in AS but also identify renal tubular immune pathways, particularly STING signaling, as promising therapeutic targets. As the use of genetic testing continues to expand in clinical practice, the recognized population of patients with AS and related kidney disease is rapidly growing. In this context, this study offers timely and compelling evidence supporting SGLT2 inhibition as a rational and potentially disease-modifying strategy in this expanding patient population.

## Supplementary Material
