# Supplementary material for: Sodium-Glucose Cotransporter 2 Inhibitors in Alport Syndrome: Emerging Clinical Evidence and Mechanistic Insights
Source: Kidney360. 2026 May 28;7(5):938–41. doi: 10.34067/KID.0000001166 (PMC13229413; doi:10.34067/KID.0000001166)
Supplement: Supplementary file 1 [file kidney360-7-0938-s001.pdf]

ASN American Society of Nephrology | JASN Journal of the American Society of Nephrology | KIDNEY360 Accessing Our World From Every Angle | CJASN Clinical Journal of the American Society of Nephrology

ASN Journal Disclosure Form

As per ASN journal policy, I have disclosed any financial relationships or commitments I have held in the past 36 months as included below. I have listed my Current Employer below to indicate there is a relationship requiring disclosure. If no relationship exists, my Current Employer is not listed.

J. Miner reports the following:
Employer: WashU Medicine Division of Nephrology; Consultancy: Bayer, Eloxx Pharmaceuticals, Sintra Therapeutics, SonoThera, Travere, Alcimed, Visterra, Gilde Healthcare Partners, Purespring Therapeutics, Nionyx Bio; Ownership Interest: Sintra Therapeutics, Inc.; Research Funding: Keros Therapeutics; Honoraria: Axiom Healthcare Strategies, Daiichi Sankyo; Patents or Royalties: Kerafast; Elsevier; Genentech; Sintra Therapeutics; SonoThera; Advisory or Leadership Role: American Society of Nephrology Treasurer and Councilor; Kidney International, Editorial Board; Journal of Clinical Investigation, Consulting Editor; Matrix Biology, Editorial Board; Matrix Biology Plus, Editorial Board; American Society for Matrix Biology, Past President; and Other Interests or Relationships: Alport Syndrome Foundation: Scientific Advisory Research Network.

I understand that the information above will be published within the journal article, if accepted, and that failure to comply and/or to accurately and completely report the potential financial conflicts of interest could lead to the following: 1) Prior to publication, article rejection, or 2) Post-publication, sanctions ranging from, but not limited to, issuing a correction, reporting the inaccurate information to the authors' institution, banning authors from submitting work to ASN journals for varying lengths of time, and/or retraction of the published work.

Name: Jeffrey H. Miner
Manuscript ID: K360-2026-000052
Manuscript Title: SGLT2 Inhibitors in Alport Syndrome: Emerging Clinical Evidence and Mechanistic Insight
Date of Completion: January 20, 2026
Disclosure Updated Date: December 18, 2025

ASN
American Society of Nephrology
JASN
Journal of the American Society of Nephrology
KIDNEY360
Accessing Our World From Every Angle
CJASN
Clinical Journal of the American Society of Nephrology

ASN Journal Disclosure Form

As per ASN journal policy, I have disclosed any financial relationships or commitments I have held in the past 36 months as included below. I have listed my Current Employer below to indicate there is a relationship requiring disclosure. If no relationship exists, my Current Employer is not listed.

K. Miyata reports the following:
Employer: Saint Louis University

I understand that the information above will be published within the journal article, if accepted, and that failure to comply and/or to accurately and completely report the potential financial conflicts of interest could lead to the following: 1) Prior to publication, article rejection, or 2) Post-publication, sanctions ranging from, but not limited to, issuing a correction, reporting the inaccurate information to the authors' institution, banning authors from submitting work to ASN journals for varying lengths of time, and/or retraction of the published work.

Name: Kana Miyata
Manuscript ID: K360-2026-000052
Manuscript Title: SGLT2 Inhibitors in Alport Syndrome: Emerging Clinical Evidence and Mechanistic Insight
Date of Completion: January 20, 2026
Disclosure Updated Date: January 20, 2026
